# Integrated transcriptomic and proteomic analyses of the mechanisms of *Citrobacter freundii*–induced ‘‘water bubble disease’’ in *Macrobrachium rosenbergii*

**DOI:** 10.3389/fimmu.2026.1894276

**Published:** 2026-10-22

**Authors:** Feifei Wang, Binbin Lv, Yanqiong Huang, Ling Wang, Chaoxiong Ren, Aiping Tan, Hua Gong, Yingtiao Lai, Fei Zhao

**Affiliations:** 1 Key Laboratory of Fishery Drug Development, Ministry Agriculture and Rural Affairs, Guangdong Provincial Key Laboratory of Aquatic Animal Immunology and Sustainable Aquaculture, Pearl River Fisheries Research Institute, Chinese Academy of Fishery Sciences, Guangzhou, China; 2 Integrated Technique & Service Center of Guangzhou Baiyun Airport Customs, State Key Testing Laboratory of Aquatic Products, Guangzhou Baiyun Airport Customs, Guangzhou, China; 3 Zhaoqing Branch Center of Guangdong Laboratory for Lingnan Modern Agricultural Science and Technology, Zhaoqing, China

**Keywords:** Citrobacter freundii, host–pathogen interaction, immune evasion, Macrobrachium rosenbergii, multi-omics, water bubble disease

## Abstract

The frequent outbreaks of “water bubble disease” in recent years have emerged as a major bottleneck hindering the healthy and sustainable development of the *Macrobrachium rosenbergii* farming industry. This study aimed to confirm the pathogenicity of *Citrobacter freundii* in *M. rosenbergii* through bacterial isolation, identification, challenge tests, and histopathological examination. The median lethal dose was determined to be 1 × 10^4^·^6^ CFU/mL. Integrated transcriptomic and proteomic analyses were conducted to further elucidate the molecular mechanisms underlying the dynamic changes in hepatopancreas at 0, 6, and 24 h following *C. freundii* infection. Progressive tissue damage was observed in the hepatopancreas, including disintegration of the basement membrane and severe cellular vacuolization. The results revealed a distinct temporal pattern in the host defense. At 6 hpi, activation of interleukin-17/nuclear factor κB–mediated inflammatory responses and DNA damage repair pathways was observed, whereas at 24 hpi, enrichment of cytoskeletal remodeling and cardiac muscle contraction pathways was detected, which may be associated with the “water bubble” phenotype. Furthermore, the omics data suggest that *C. freundii* may employ multilayered immune evasion strategies. At 6 hpi, antigen presentation and lysosomal functions were suppressed, potentially facilitating immune evasion. At 24 hpi, host clearance mechanisms such as phagocytosis, autophagy, and apoptosis were inhibited, coupled with alterations in host energy metabolism, which may support bacterial survival. Transcript–protein correlation analysis further indicated a shift from “decoupling” to “recoupling” in host gene expression regulation over the course of infection. This study provides a multi-omics-based systematic analysis of the molecular changes associated with “water bubble disease” in *M. rosenbergii*. It offers insights into the potential pathogenesis of *C. freundii* and provides a foundation for developing targeted control strategies focused on immune-metabolic pathways.

## 1 Introduction

The giant freshwater prawn (*Macrobrachium rosenbergii*) is a key species in global freshwater aquaculture, with rapid expansion and technological advancement in its farming industry (1). However, the widespread adoption of high-density intensive farming has increased the incidence of disease outbreaks caused by ecosystem imbalances (2). Multiple stressors, including environmental factors (e.g., deteriorating water quality and temperature fluctuations), bacterial pathogens (e.g., *Aeromonas* spp. and *Vibrio* spp.), and viral pathogens (e.g., iridovirus and white spot syndrome virus), contribute to persistently high mortality in adult prawns, severely constraining the sustainable development of the industry.

A disease known as “water bubble disease” has been increasingly reported in *M. rosenbergii* farming in recent years, posing a major threat to industry development (3). Based on our preliminary epidemiological investigation, the disease exhibits a typical progressive course characterized by distinct pathological changes. The disease can be divided into three stages. Early stage (~2 days): *M. rosenbergii* exhibits reduced feeding and decreased locomotor activity, accompanied by a marked decline in vitality. Middle stage (~3 days): Transparent vesicles (~5 mm in diameter) emerge at the junction between the cephalothorax and hepatopancreas. Concurrently, the hepatopancreas undergoes progressive atrophy, and discoloration, changing from the normal yellowish-brown color to dark red. Terminal stage (>4 days): The vesicles progressively enlarge and may eventually occupy the entire tissue space between the cephalothorax and the hepatopancreas–stomach region. Simultaneously, the hepatopancreas tissue undergoes necrosis and liquefaction, manifesting as whitish decay with a putrid odor. These changes are accompanied by gastric and intestinal emptying, locomotor impairment, and eventual mortality. These pathological changes not only impair individual growth and survival but also threaten the overall health of farmed populations.

Etiological studies have identified *Citrobacter freundii* as the primary causative agent of “water bubble disease” (3, 4). This opportunistic pathogen exhibits high genomic plasticity, enabling rapid acquisition of virulence factors and antimicrobial resistance (5, 6). It causes nosocomial infections (e.g., urinary tract infections, bacteremia, and neonatal meningitis) and produces inducible AmpC β-lactamase in mammals, contributing to multidrug resistance (5). *C. freundii* has emerged as a major pathogen causing substantial economic losses in aquatic animals. It induces hemorrhagic enteritis and septicemia in fish, with transcriptomic analysis revealing manipulation of host metabolic and immune pathways (e.g., ubiquitin-mediated proteolysis and phagosomes) (7). In crustaceans, *C. freundii* has been linked to mass mortalities in prawns, crayfish, and crabs, causing severe damage to the hepatopancreas and intestines, and activating immune-related genes (e.g., MyD88 and proPO) (4, 8, 9). Recently, researchers identified *C. freundii* as the primary etiological agent of “water bubble disease” in *M. rosenbergii* and evaluated antibiotic efficacy, documenting changes in antioxidant enzyme activities and the expression of immune-related genes following infection (3). Moreover, a highly virulent *C. freundii* strain was isolated from diseased *M. rosenbergii*, and its virulence-related genes and enzymatic activities were characterized; the temporal expression of immune-related genes in multiple tissues was also monitored (4). Despite these contributions, previous studies have largely focused on pathogen isolation, antibiotic susceptibility testing, and targeted assessment of immune parameters measurements, without systematically characterizing host–pathogen interactions at the global transcriptional and translational levels, or elucidating the dynamic interplay between host defenses and pathogen-mediated immune evasion during infection.

Therefore, this study was conducted to investigate the pathogenicity of *C. freundii* in *M. rosenbergii* through histopathological examination, pathogen isolation and purification, and challenge experiments, and to determine its median lethal dose (LD_50_). We then integrated transcriptomic and proteomic approaches to systematically analyze dynamic changes in gene expression and protein translation in the hepatopancreas at 0, 6, and 24 h post-infection (hpi). Through functional annotation and pathway enrichment analysis of differentially expressed genes (DEGs) and proteins, this study aimed to: (1) characterize early host immune responses, particularly the activation of inflammatory signaling and DNA damage repair pathways; (2) elucidate alterations in host muscle structure, energy metabolism, and cellular homeostasis systems during the mid-to-late stages of infection; and (3) uncover how *C. freundii* evades host immunity by interfering with antigen presentation, phagocytosis, autophagy, and apoptosis to sustain its intracellular survival. Finally, by correlating multi-omics data, we aimed to construct a time-resolved regulatory network of host–pathogen interactions during infection, providing a theoretical foundation for understanding the molecular basis of *C. freundii* pathogenicity and developing targeted disease prevention and control strategies.

## 2 Materials and methods

### 2.1 Isolation and identification of pathogenic bacteria

*M. rosenbergii* that were naturally diseased with obvious disease characteristics were randomly selected from a prawn aquaculture farm in Zhaoqing City, Guangdong Province, China, and disinfected with 75% ethanol before dissection.Using sterile scissors and forceps, the cephalothoracic armor of *M. rosenbergii* was dissected to expose the water bubble. The blister fluid from the water bubble was aseptically aspirated via a sterile syringe. Concurrently, tissue specimens from the hepatopancreas and gills were excised. A volume of 500 µL of sterile phosphate-buffered saline (PBS) was added to all collected samples for tissue homogenization at 4°C. Subsequently, 100 µL of the resulting homogenate was plated onto brain heart infusion (BHI) agar medium and incubated at 28°C for 16−18 h. Predominant bacterial colonies were selected for isolation and purification, and subjected to molecular identification via 16S rRNA gene sequencing.

### 2.2 Recurrent infection and sample collection

Healthy *M. rosenbergii* (mean body weight: 10.00 ± 0.50 g) individuals were obtained from Liantang Town, Zhaoqing City, Guangdong Province, China. Upon arrival, the prawns were acclimated in a 2-m³ aerated tank maintained at 28 °C ± 2 °C and a pH of 7.5–8.0, and fed a commercial diet three times daily. Following a 2-week acclimation period, healthy, uniformly sized individuals that tested negative for common pathogens were selected for subsequent experiments.

A total of 180 healthy *M. rosenbergii* (mean body weight: 10 g) individuals were randomly assigned into 6 groups (*n* = 30 per group), including 5 treatment groups and one control group. Each treatment group received an intramuscular injection of 10 μL of *C. freundii* suspension at concentrations of 1.0 × 10^4^, 1.0 × 10^5^, 1.0 × 10^6^, 1.0 × 10^7^, or 1.0 × 10^8^ CFU/mL, respectively. The control group was administered an equivalent volume of PBS. The survival of *M. rosenbergii* isolates was recorded at 150 hpi, and the LD_50_ was determined at 24 hpi.

Additionally, 60 healthy *M. rosenbergii* individuals were randomly assigned to a PBS stimulation group (PBS) and a *C. freundii* stimulation group (CF). Based on the calculated LD_50_ (1 × 10^4^·^6^ CFU/mL), prawns in the CF group were intramuscularly injected at the second abdominal segment with 10 μL of a bacterial suspension, whereas prawns in the PBS group were injected with 10 μL of PBS. Prior to hepatopancreas collection at each time point (0, 6, and 24 hpi), prawns were euthanized by rapid cooling on ice (approximately 1 °C–4 °C) for 10–15 min until complete loss of appendage movement and the righting reflex, followed by immediate decapitation with sterile scissors. Hepatopancreas tissues were then collected using sterile forceps and scissors. Three biological replicates were used at each time point, with each replicate consisting of hepatopancreas tissues pooled from three individual prawns. Portions of the hepatopancreatic tissue were placed in sterile centrifuge tubes pre-filled with 4% paraformaldehyde for tissue fixation, whereas the remaining samples were flash-frozen in liquid nitrogen and stored at −80 °C for subsequent RNA and protein extraction.

### 2.3 Hematoxylin and eosin staining

The hepatopancreatic tissues fixed in 4% paraformaldehyde were dehydrated through a graded ethanol series, cleared in xylene, embedded in paraffin, and sectioned at a thickness of 4–5 μm. After deparaffinization and rehydration, the sections were stained with hematoxylin for 5–8 min, rinsed with running water, differentiated in 1% acid ethanol, blued, and counterstained with eosin for 2–3 min. The stained sections were then dehydrated, cleared, mounted with neutral balsam, and examined under a light microscope (Carl Zeiss, Germany) for image acquisition.

### 2.4 Transcriptome sequencing and analysis

Total RNA was extracted from hepatopancreas samples collected at 0, 6, and 24 hpi using TRIzol reagent following the manufacturer’s protocols, with the 0 hpi samples collected from the PBS group at 0 h post-injection and serving as the baseline control. RNA integrity and concentration were assessed using an Agilent 2100 Bioanalyzer and NanoDrop spectrophotometer, respectively, and high-quality RNA samples were used for library construction. mRNA was enriched using mRNA Capture Beads, purified, and fragmented at a high temperature. The fragmented mRNA served as the template to synthesize the first strand of cDNA in a reverse transcription enzyme mix, followed by synthesis of the second cDNA strand, with simultaneous end repair and A-tailing. Adapters were then ligated, and target fragments were selected and purified using Hieff NGS DNA Selection Beads. Subsequently, PCR amplification was performed for library construction, and final detection was carried out on the Illumina NovaSeq X Plus.

Raw sequencing data were quality-filtered using Fastp to remove adapters and low-quality reads (10). Clean reads were aligned to the *M. rosenbergii* reference genome using HISAT2 (11).The gene expression levels were quantified as Fragments Per Kilobase of transcript per Million mapped reads (FPKM) using StringTie v1.3.1 (12, 13). Differential expression analysis between groups (0 h vs 6 h, 0 h vs 24 h, and 6 h vs 24 h) was performed using the DESeq2 package in R (14, 15), with genes meeting the criteria of |log_2_FoldChange| >1 and adjusted *P* value <0.05 defined as DEGs. Gene Ontology (GO) and Kyoto Encyclopedia of Genes and Genomes (KEGG) pathway enrichment analyses of DEGs were conducted using the clusterProfiler package, with corrected *P* value <0.05 indicating significant enrichment (16, 17). Gene set enrichment analysis (GSEA) was performed to identify pathways with coordinated expression changes (18). Protein–protein interaction (PPI) networks of key DEGs were constructed using the STRING database and visualized in Cytoscape to identify hub genes (19).

### 2.5 Proteomics sequencing and analysis

Hepatopancreas tissues were homogenized in lysis buffer for total protein extraction, and protein concentration was determined using a BCA assay. Samples were then processed through denaturation, reduction, alkylation, tryptic digestion, and peptide cleanup. The samples were analyzed using a Bruker timsTOF Pro 2 mass spectrometer, with data-dependent acquisition used for library construction and data-independent acquisition (DIA) for protein identification and quantification. DIA data were processed and analyzed using Spectronaut 18 (Biognosys AG, Switzerland) with default settings.

Protein functions and classifications were annotated using the GO, EuKaryotic Orthologous Group (KOG)/Cluster of Orthologous Groups of Proteins (COG), and the KEGG databases (20). Venn diagrams were used to illustrate gene distribution and database matching results. Principal component analysis (PCA) was performed in R (http://www.r-project.org/) to assess variations in protein expression profiles. Differentially expressed proteins (DEPs) were identified using thresholds of absolute fold change >1.5 and a Q-value (adjusted *P* value) < 0.05. These significant proteins were subsequently subjected to GO and KEGG enrichment analyses. PPI networks were constructed based on the STRING database (http://string-db.org). Moreover, GSEA was conducted using GSEA software and the MSigDB database to identify significantly enriched signaling pathways.

### 2.6 Correlation analysis of transcriptomic and proteomic data

Integrated analysis of transcriptomic and proteomic data was performed to investigate the relationship between transcriptional and translational regulation during infection. DEGs/DEPs identified in the same comparison groups (0 h vs 6 h, 0 h vs 24 h, and 6 h vs 24 h) were overlapped using Venn diagrams to identify consistently regulated entities. The correlation between mRNA expression (FPKM) and protein abundance (intensity) for each paired sample was assessed using Pearson correlation coefficients. Molecules were categorized based on their differential expression patterns at the transcript and protein levels using a nine-quadrant analysis. Molecules in key quadrants were subsequently subjected to KEGG pathway enrichment analysis to identify biologically coherent regulatory modules. Heatmaps were generated to visualize the expression dynamics of key overlapping molecules across time points at both omics layers.

### 2.7 Quantitative real-time PCR analysis

To investigate the transcriptional dynamics of core host defense genes in *M. rosenbergii*, quantitative reverse transcription–polymerase chain reaction (qRT-PCR) assays were performed. The elongation factor (EF) gene was employed as the endogenous reference gene for data normalization. The primer sequences used for all target and reference genes are listed in **Supplementary Table S1**. Amplification reactions were performed using a 7500 Real-Time PCR System (Applied Biosystems, CA, USA). Relative transcript levels of the target genes were calculated using the 2^−ΔΔCt^ method (21). Graphical representation of the obtained data was generated using GraphPad Prism 8.0.Statistical significance was determined by one-way ANOVA followed by Tukey’s *post hoc test*; *P* < 0.05.

## 3 Results

### 3.1 Identification and pathogenicity of *C. freundii* in *M. rosenbergii*

In this study, we systematically investigated the etiology and histopathology of diseased freshwater prawns (*M. rosenbergii*). The sample shown in **Figure 1A** was collected from a naturally diseased *M. rosenbergii* individual exhibiting typical clinical signs of “water bubble disease” during an outbreak at a prawn aquaculture farm in Zhaoqing City, Guangdong Province, China, in October 2025. Pathological examination revealed that including a translucent vesicle (approximately 5 mm in diameter) at the junction of the cephalothorax and hepatopancreas, filled with clear fluid, along with a distinct reddish discoloration of the hepatopancreatic tissue. Pathogen isolation and purification of the vesicular fluid and hepatopancreatic tissue were performed. Morphological and molecular analyses identified *C. freundii* as the dominant bacterial isolate. The 16S rRNA gene sequencing revealed 100% homology with known reference strains of *C. freundii* (**Figure 1B**). Pathogenicity was confirmed through a challenge experiment. The results demonstrated that the *C. freundii* isolate exerted a concentration-dependent pathogenic effect on *M. rosenbergii*, with higher doses leading to progressively decreased survival. The 24-h LD_50_ was determined to be 1 × 10^4^·^6^ CFU/mL using the Karber method based on mortality at 24 hpi. At this time point, transparent vesicle formation at the cephalothorax–hepatopancreas junction was already clearly observable. In contrast, cumulative survival was monitored up to 150 hpi to capture the complete mortality curve, high-dose infection groups (≥10^5^ CFU/mL) exhibited cumulative survival rates as low as 20%, indicating strong virulence of the isolated strain (**Figure 1C**). Histopathological examination revealed progressive hepatopancreatic damage, including basement membrane disintegration and severe cellular vacuolization (**Figure 1D**). These findings confirmed *C. freundii* as the primary etiological agent responsible for the “water bubble disease” in *M. rosenbergii*.

**Figure 1:**
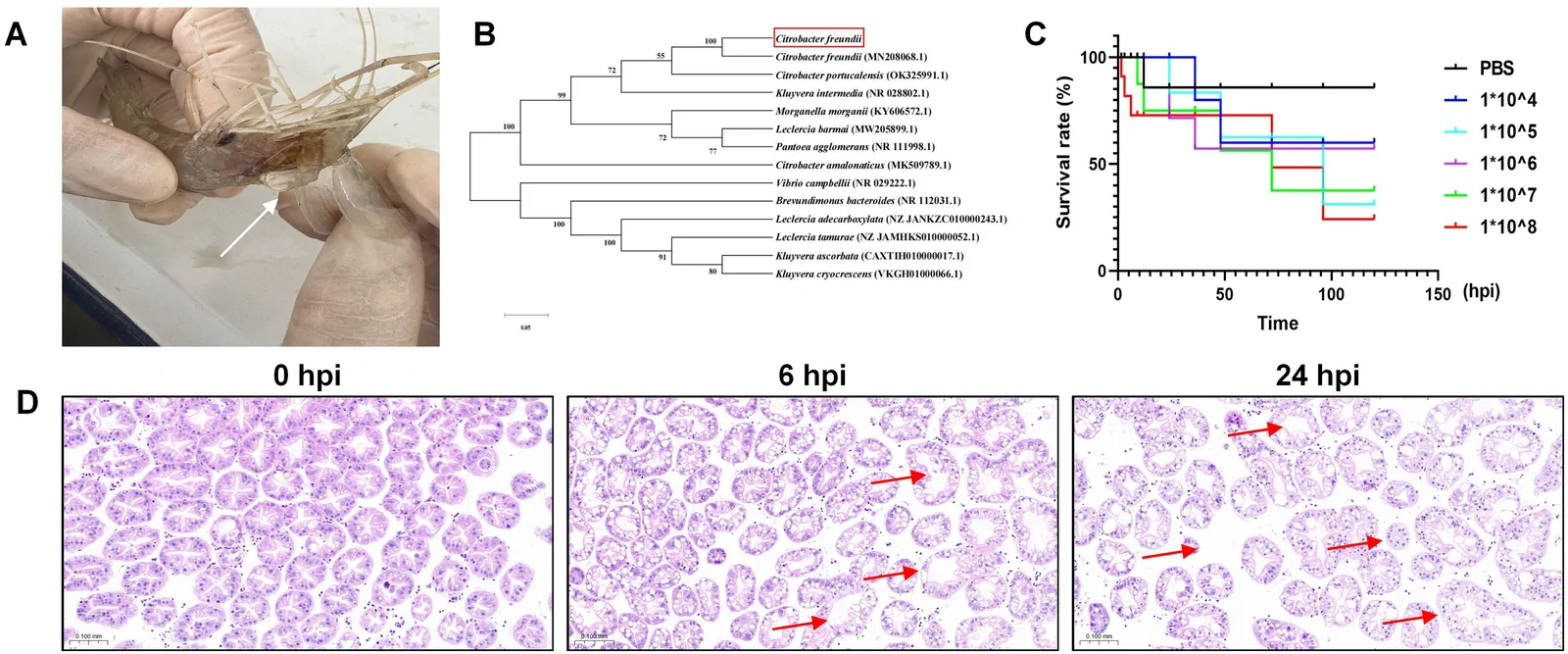
Clinical symptoms and pathogenic identification of “water bubble disease” in *M. rosenbergii*. **(A)** Representative clinical symptoms of *M. rosenbergii* affected by “water bubble disease”. The specimen was collected from a naturally diseased individual during an outbreak at a farm in Zhaoqing City, Guangdong Province, China, in October 2025; the photograph was taken immediately after collection, prior to dissection for pathogen isolation. The white arrow indicates a transparent vesicle located at the junction of the carapace and the hepatopancreas. **(B)** Phylogenetic tree of *C. freundii* based on 16S rRNA analysis. The tree was constructed using the neighbor-joining method with 1000 bootstrap replicates; bootstrap values (>50%) are shown at the nodes. **(C)** Survival curves of *M. rosenbergii* following injection with different concentrations of *C. freundii*. (1.0 × 10^4^ to 1.0 × 10^8^ CFU/mL). **(D)** Histopathological changes in the hepatopancreas of *M. rosenbergii* infected with *C. freundii*. Red arrows indicate hepatopancreatic cell damage or vacuolation. Scale bar: 0.100 mm.

### 3.2 Transcriptomic profiling

#### 3.2.1 Quality control and expression patterns of mRNA-seq data

We performed transcriptome sequencing at 0, 6, and 24 hpi which respectively represent pre-infection, early infection, and the later stage of infection (the latter being defined relative to 6 hpi) to investigate the transcriptional response of the hepatopancreas in *M. rosenbergii* following *C. freundii* infection. The sequencing data were of high quality, with clean reads >99.58%, total mapping rate >92.69%, and exon coverage >82% across all samples. In total, 24, 835 genes were identified, comprising 23, 057 known genes and 1778 newly predicted genes (**Table 1**). Venn diagram analysis revealed that 7940 genes were shared across all time points (**Supplementary Figure S1A**). PCA demonstrated strong reproducibility among biological replicates and clear separation between different time points (**Supplementary Figure S1B**), indicating time as a key driver of gene expression changes. Differential expression analysis identified 2530, 4256, and 1434 DEGs in the 0 h vs 6 h, 0 h vs 24 h, and 6 h vs 24 h comparison groups, respectively, with 2290, 3857, and 1025 upregulated genes (**Supplementary Figure S1C**). Notably, 576 DEGs were shared among all comparison groups (**Supplementary Figure S1D**), suggesting central regulatory roles of these genes in bacterial challenge response. These results indicated that *C. freundii* stimulation markedly altered the hepatopancreatic transcriptome of *M. rosenbergii* over time, highlighting a core set of persistently responsive genes and providing important insights into the molecular mechanisms underlying host immune response.

**Table 1:** Basic information of transcriptome sequencing.

Sample	RawDatas	Clean_Reads(%)	Unmapped_Reads(%)	Total_Mapped(%)	Exon	Refer_Genes	Novel_Genes	Total_Genes	sequenced_Total_Genes(%)
CF-0h-1	42215130	42055520 (99.62%)	39256176 ( 93.34% )	36764699 (93.65%)	30919239 (84.10%)	23057	1778	24835	13328 (53.67%)
CF-0h-2	42092010	41948824 (99.66%)	39139388 ( 93.30% )	36661951 (93.67%)	30813070 (84.05%)	23057	1778	24835	13123 (52.84%)
CF-0h-3	41228690	41082076 (99.64%)	38289008 ( 93.20% )	35760250 (93.40%)	29973862 (83.82%)	23057	1778	24835	13080 (52.67%)
CF-6h-1	42256746	42090498 (99.61%)	38263730 ( 90.91% )	35626615 (93.11%)	29371539 (82.44%)	23057	1778	24835	14518 (58.46%)
CF-6h-2	44604162	44443644 (99.64%)	41590700 ( 93.58% )	38937298 (93.62%)	32330555 (83.03%)	23057	1778	24835	14971 (60.28%)
CF-6h-3	44576750	44409134 (99.62%)	39565280 ( 89.09% )	36871202 (93.19%)	30299655 (82.18%)	23057	1778	24835	14357 (57.81%)
CF-24h-1	44790784	44603468 (99.58%)	39779098 ( 89.18% )	36947997 (92.88%)	30581642 (82.77%)	23057	1778	24835	14901 (60.00%)
CF-24h-2	43250352	43083028 (99.61%)	39224964 ( 91.05% )	36628109 (93.38%)	30462231 (83.17%)	23057	1778	24835	15149 (61.00%)
CF-24h-3	45609474	45485020 (99.73%)	40670628 ( 89.42% )	37698008 (92.69%)	31180766 (82.71%)	23057	1778	24835	14885 (59.94%)

#### 3.2.2 GO enrichment analysis of DEGs

To further elucidate the functional landscape of DEGs in response to *C. freundii* infection, GO enrichment analysis was performed for three comparison groups: 0 h vs 6 h, 0 h vs 24 h, and 6 h vs 24 h. A broad overview of GO categories revealed that the most enriched biological processes across all time points included cellular process, metabolic process, regulation of biological process, and response to stimulus (**Figure 2A**). At the molecular function level, binding and catalytic activity were consistently overrepresented whereas the cellular component terms were associated with cellular anatomical entity and protein-containing complexes (**Figure 2A**). These results suggested coordinated transcriptional reprogramming in the hepatopancreas, involving fundamental cellular functions, regulatory pathways, and structural reorganization following infection.

**Figure 2:**
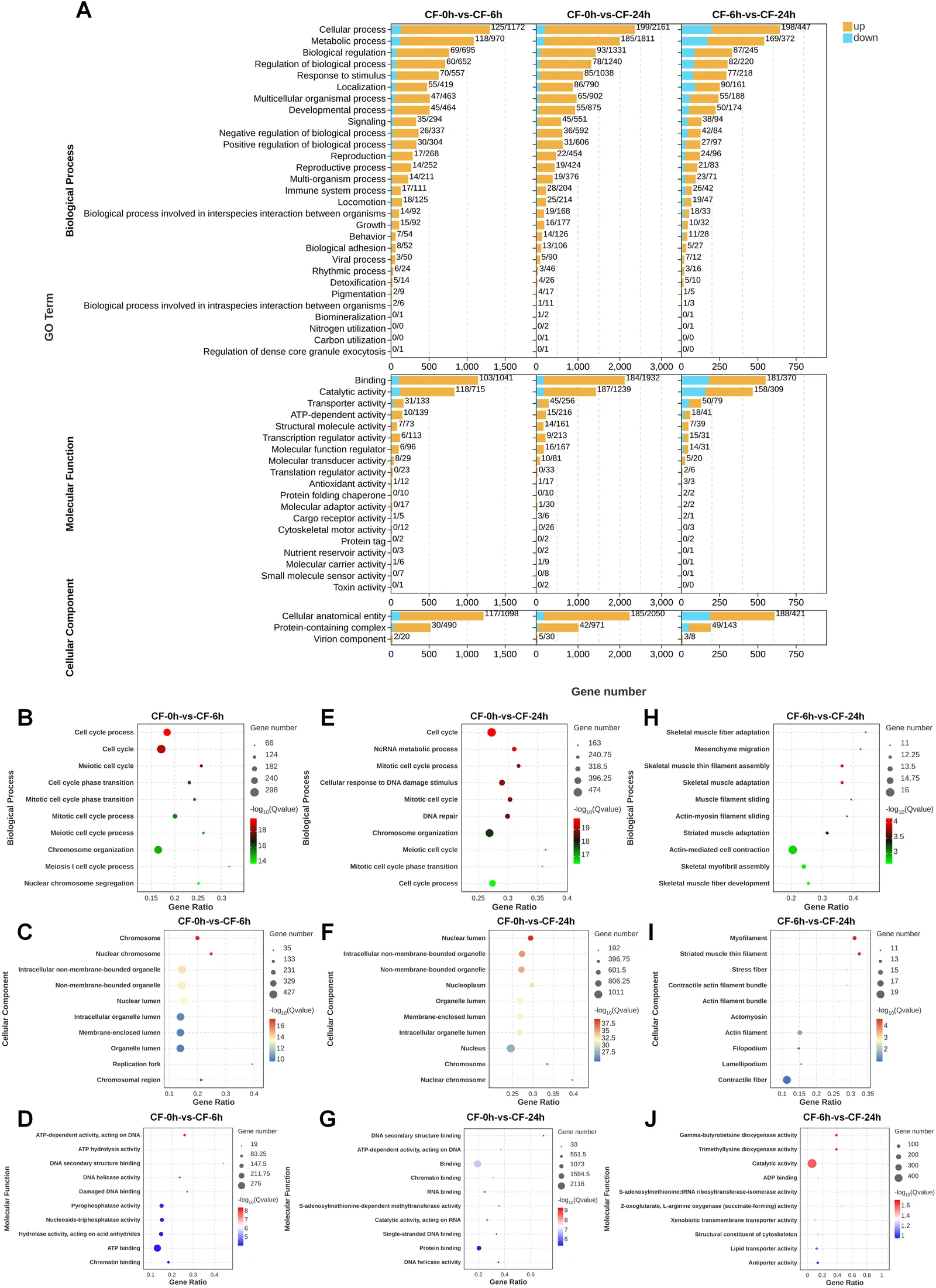
Functional analysis of DEGs in *M. rosenbergii* following *C. freundii* infection, based on GO enrichment analysis. **(A)** GO enrichment analysis of DEGs across all comparison groups. **(B, E, H)** Top 10 enriched biological processes of DEGs in the 0 h vs 6 h, 0 h vs 24 h, and 6 h vs 24 h comparison groups, respectively. **(C, F, I)** Top 10 enriched cellular components of DEGs in the respective comparison groups. **(D, G, J)** Top 10 enriched molecular functions of DEGs in the respective comparison groups.

In the 0 h vs 6 h comparison group, the enriched biological processes were mainly associated with cell cycle regulation, including “cell cycle process, “ “meiotic/mitotic cell cycle, “ and “chromosome organization” (**Figure 2B**). Corresponding cellular components were predominantly associated with nuclear and chromosomal structures, such as “chromosome, “ “nuclear chromosome, “ and “replication fork” (**Figure 2C**). At the molecular function level, DNA- and ATP-related activities, such as “ATP-dependent activity, acting on DNA, “ “DNA helicase activity, “ and “damaged DNA binding, “ were prominent (**Figure 2D**). This pattern indicated an early activation of DNA replication, repair, and cell cycle checkpoints following infection. In the 0 h vs 24 h comparison group, the functional profile expanded. While cell cycle–related processes remained enriched, additional terms such as “ncRNA metabolic process, “ “cellular response to DNA damage stimulus, “ and “DNA repair” became prominent (**Figure 2E**). Cellular components continued to highlight nuclear compartments such as “nuclear lumen” and “nucleoplasm” (**Figure 2F**). Molecular functions expanded to include not only DNA-related activities such as “DNA secondary structure binding, “ but also RNA and chromatin modulation such as “RNA binding, “ “chromatin binding, “ and “catalytic activity, acting on RNA” (**Figure 2G**). This shift suggested a progression from immediate cell cycle control to more sustained responses, including nucleic acid metabolism, damage repair, and epigenetic or post-transcriptional regulation at the 24-h time point. The 6 h vs 24 h comparison group revealed a distinct functional transition. The enriched biological processes were overwhelmingly related to muscle structure and function, including “skeletal muscle fiber adaptation, “ “mesenchyme migration, “ “actin-myosin filament sliding, “ and various muscle filament assembly terms (**Figure 2H**). Consistently, cellular components were enriched in cytoskeletal and contractile structures such as “myofilament, “ “actin filament bundle, “ “stress fiber, “ and “filopodium” (**Figure 2I**). At the molecular function level, terms shifted toward enzymatic activities related to muscle metabolism and transport, such as specific dioxygenase activities (e.g., gamma-butyrobetaine dioxygenase activity), “structural constituent of cytoskeleton, “ and various transporter activities (**Figure 2J**). This pattern indicated a major transcriptional reprogramming in the hepatopancreas toward cytoskeletal remodeling, muscle adaptation, and associated metabolic changes during the later phase of infection (6 h vs 24 h).

The GO enrichment analysis revealed a dynamic, stage-specific transcriptional response in the hepatopancreas of *M. rosenbergii* to *C. freundii* infection. The early response (0 h vs 6 h) was characterized by cell cycle and DNA metabolism activities, possibly indicating cellular preparation or stress response. The mid-to-late response (0 h vs 24 h) expanded to include DNA repair, RNA processing, and broader regulatory mechanisms. Most notably, the later-stage comparison (6 h vs 24 h) revealed a strong shift toward muscle-related processes and cytoskeletal reorganization, indicating significant structural and functional adaptation within the hepatopancreas tissue during infection.

#### 3.2.3 KEGG pathway enrichment analysis of DEGs

We performed KEGG pathway enrichment analysis to further elucidate the functional dynamics of DEGs in *M. rosenbergii* during *C. freundii* infection. In the early infection stage (0 h vs 6 h), the top 20 enriched pathways were significantly associated with DNA repair and cell cycle–related signaling pathways, including the Fanconi anemia pathway, cell cycle, mismatch repair, DNA replication, and nucleotide excision repair. Notably, the IL-17 signaling pathway was also significantly enriched, indicating rapid activation of inflammatory defense responses during early infection (**Figure 3A**). GSEA further confirmed that these pathways were significantly upregulated at 6 hpi (**Figure 3B**). Key genes within these pathways remained consistently upregulated at both 6 and 24 hpi (**Figure 3C**), suggesting that the host’s primary response to pathogen recognition is to activate mechanisms that protect genomic integrity and maintain stability. A PPI network was constructed to identify key regulatory nodes within the interaction network. The results revealed that at 6 hpi, HSP90A, PCNA, and TFDP2 genes exhibited significant differences in both connectivity and expression levels (**Figure 3D**), indicating central regulatory roles of these molecules in the early immune response of *M. rosenbergii* to *C. freundii* infection.

**Figure 3:**
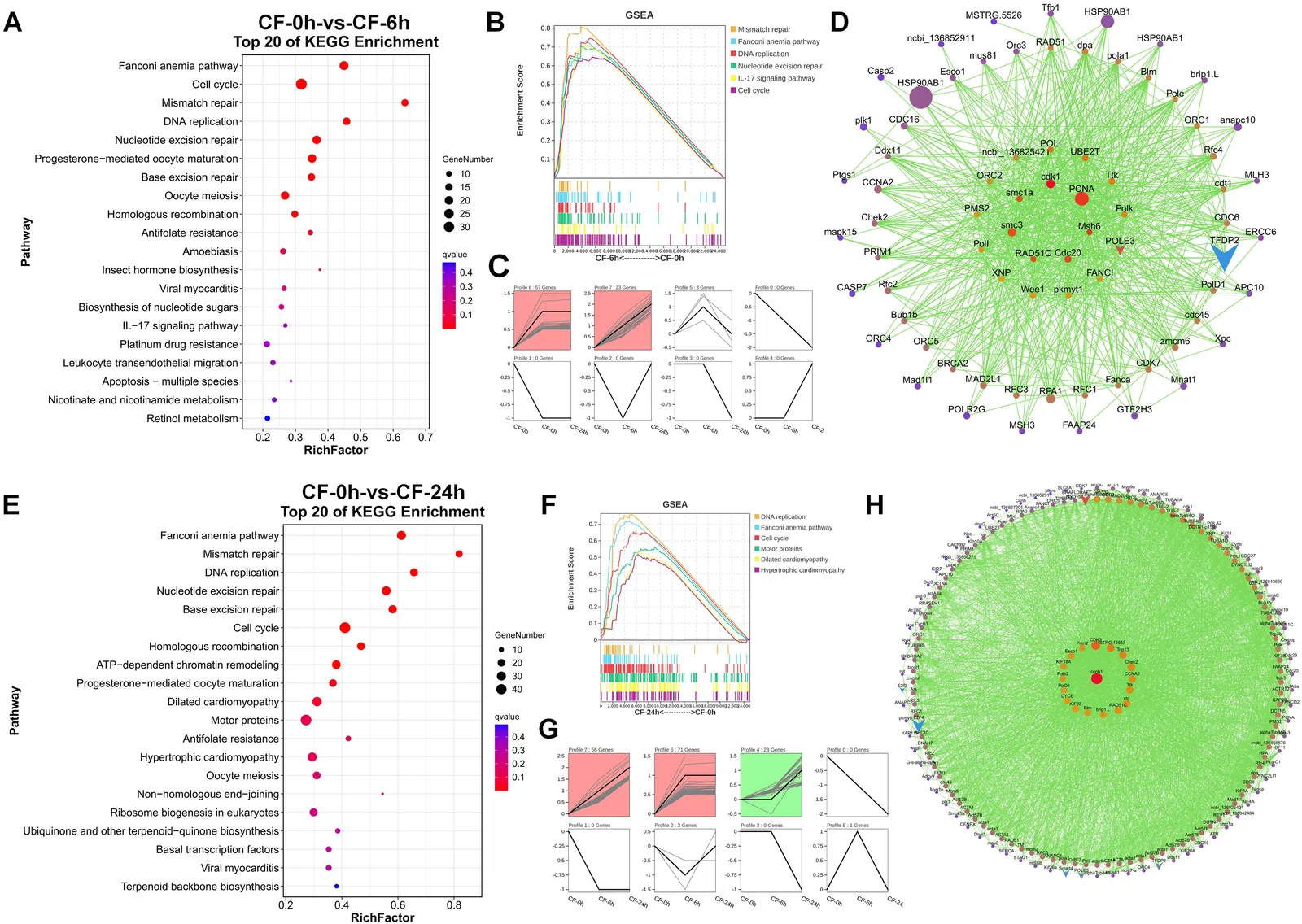
Functional analysis of DEGs in *M. rosenbergii* following *C. freundii* infection based on KEGG enrichment analysis. **(A)** KEGG enrichment analysis of DEGs at 6 hpi with *C. freundii* injection. **(B)** GSEA of pathways related to DNA damage repair and inflammatory signaling. **(C)** Temporal expression trends of DEGs involved in DNA damage repair and inflammation-related signaling pathways. **(D)** PPI network analysis of DEGs within DNA damage repair and inflammatory signaling pathways at 6 hpi. Nodes represent genes, with color indicating connectivity degree (redder nodes indicate higher connectivity) and size reflecting expression level. **(E)** KEGG enrichment analysis of DEGs at 24 hpi with *C. freundii* infection. **(F)** GSEA of signaling pathways associated with tissue damage. **(G)** Trend analysis of DEGs involved in tissue damage–related signaling pathways. **(H)** PPI network analysis of DEGs within tissue damage–related signaling pathways at 24 hpi. Nodes represent genes, with color indicating connectivity degree (nodes with deep red coloration indicate higher connectivity) and size reflecting expression level.

As the infection progressed to 24 h (0 h vs 24 h), DNA repair–related pathways, such as the Fanconi anemia pathway, cell cycle, and DNA replication, remained among the top 20 enriched pathways, indicating sustained genomic stability. Concurrently, a notable shift in profile enrichment was observed, with cardiomuscular-related pathways, such as dilated cardiomyopathy, hypertrophic cardiomyopathy, and motor proteins, becoming significantly enriched (**Figure 3E**). GSEA confirmed significant upregulation of these pathways at 24 hpi (**Figure 3F**), and key genes within them exhibited sustained upregulation at both 6 and 24 h (**Figure 3G**). PPI network analysis further identified 18 genes, including ccnb1, CDK3, and STRG, which were hypothesized to perform core regulatory functions during the 24-h infection stage (**Figure 3H**). These findings suggest that prolonged infection leads to a shift from protective responses toward an immune-associated pathological alteration.

Notably, pathways related to muscle structure and contraction, such as cardiac muscle contraction, dilated cardiomyopathy, and adrenergic signaling in cardiomyocytes were significantly enriched and upregulated in the 6 h vs 24 h comparison (**Supplementary Figure S2**). These terms may reflect shared cytoskeletal/actin-related genes rather than direct evidence of myocardial pathology. We therefore speculate that these cytoskeletal/actin-related processes may be associated with the formation of “water bubbles” during the later stages of infection.

### 3.3 Proteomic analysis

#### 3.3.1 Quality control and quantification of protein expression

We performed proteomic sequencing to investigate the protein regulatory mechanisms in *M. rosenbergii* during *C. freundii* infection. Functional annotation of identified peptides using the COG, GO, and KEGG databases yielded 2267, 2677, and 1454 annotated proteins, respectively (**Supplementary Table S2**), of which 1361 were shared across all 3 databases (**Supplementary Figure S3A**). A total of 2733 proteins were identified across the 0-h, 6-h, and 24-h experimental groups (**Supplementary Figure S3B**). PCA demonstrated strong clustering among biological replicates within each group, indicating high reproducibility and reliability of the experimental data (**Supplementary Figure S3C**).

Differential protein expression analysis revealed 244, 382, and 546 upregulated proteins, along with 144, 109, and 209 downregulated proteins, in the 0 h vs 6 h, 0 h vs 24 h, and 6 h vs 24 h comparison groups, respectively (**Supplementary Figure S3D**). Venn diagram analysis further identified 48 proteins differentially expressed across all the 3 comparison groups (**Supplementary Figure S3E**), suggesting sustained and crucial regulatory roles of these proteins in the host response to *C. freundii* infection.

#### 3.3.2 GO enrichment analysis of DEPs

We performed GO enrichment analysis across the 0 h vs 6 h, 0 h vs 24 h, and 6 h vs 24 h comparison groups to further elucidate the biological functions of DEPs. Overall, the GO enrichment patterns at the proteomic level closely mirrored those observed at the transcriptomic level (**Figure 4A**). In terms of biological processes, DEPs were predominantly enriched in cellular process, metabolic process, biological regulation, response to stimulus, and regulation of biological process. At the molecular function level, binding and catalytic activity were predominant. Regarding cellular components, DEPs were primarily associated with cellular anatomical entities and protein-containing complexes (**Figure 4A**). This consistency indicated that, at the post-transcriptional level, the host response to pathogen infection similarly targeted core biological functions, including cellular activity, metabolic regulation, and signal transduction.

**Figure 4:**
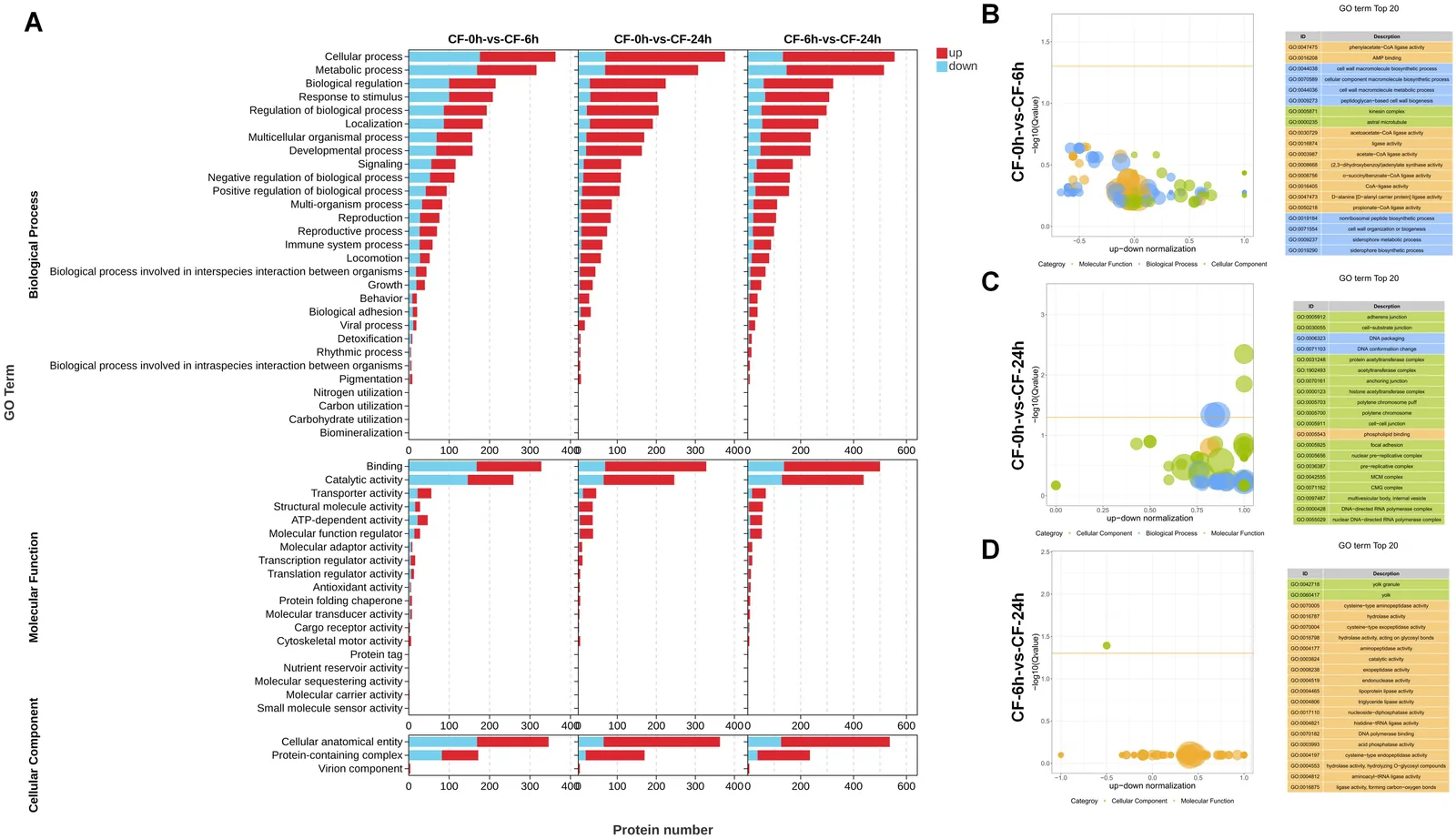
Functional analysis of DEPs in *M. rosenbergii* following *C. freundii* infection, based on GO enrichment analysis. **(A)** GO enrichment analysis of DEPs across all comparison groups (biological process, cellular component, and molecular function). **(B–D)** Top 20 enriched GO terms at 0 h vs 6 h, 0 h vs 24 h, and 6 h vs 24 h, respectively.

Further analysis of the top 20 enriched GO terms within each comparison group revealed clear temporal shifts in protein regulation and function. In the 0 h vs 6 h comparison group, DEPs were significantly enriched in cell wall synthesis and modification, such as “cell wall macromolecule biosynthetic process” and “peptidoglycan-based cell wall biogenesis.” Additionally, functions associated with secondary metabolism and microbial competition, such as “phenylacetate-CoA ligase activity” and “siderophore metabolic process, “ were also activated (**Figure 4B**). These results suggested that, during the early stage of infection, the host modulated metabolism to counter bacterial invasion. In the 0 h vs 24 h comparison group, GO enrichment shifted toward cell junction and chromatin regulation. Terms such as “adherens junction, “ “cell-substrate junction, “ “DNA packaging, “ and “histone acetyltransferase complex” were significantly enriched (**Figure 4C**), indicating enhanced cellular structural integrity, chromatin state modulation, and transcriptional regulation during infection. In the 6 h vs 24 h comparison group, DEPs were enriched in yolk metabolism and hydrolase activity, including “yolk granule, “ “cysteine-type aminopeptidase activity, “ and “triglyceride lipase activity” (**Figure 4D**). This suggested that, during the middle-to-late stages of infection, the host’s energy reserve metabolism and protein-degradation mechanisms underwent significant adjustments, possibly reflecting metabolic reallocation and energy mobilization under sustained infection pressure. These proteomic findings not only confirmed the consistency of functional responses observed in the transcriptome but also provided further insights into the host’s multilayered dynamic adaptation during *C. freundii* infection from a protein expression and modification perspective. Over time, the response shifted from structural defense and junction/epigenetic regulation toward metabolic adaptation and energy regulation.

#### 3.3.3 KEGG pathway enrichment analysis of DEPs

KEGG enrichment analysis of DEPs further revealed key signaling and metabolic responses during *C. freundii* infection. In the early infection stage (0 h vs 6 h), innate immune-related pathways, including the JAK–STAT signaling pathway, MAPK signaling pathway, and NF-κB signaling pathway, were significantly enriched (**Figure 5A**). PPI network analysis showed that HSC71, 14-3-3zeta, STAT5B, Rel, SOD2, and MAPK1 proteins exhibited high connectivity and expression levels within the network (**Figure 5B**), suggesting their central roles in early immune regulation. Heatmap analysis further confirmed that proteins including Rel, STAT5B, MAPK1, and ataxia telangiectasia mutated (ATM) were significantly upregulated during early infection, whereas HSC71, 14-3-3zeta, PKC53E, and SOD2 were notably downregulated (**Figure 5C**). Notably, cellular energy metabolism pathways, namely oxidative phosphorylation and thermogenesis, were significantly enriched in the early infection stage, and GSEA analysis indicated significant downregulation of both pathways (**Figure 5D**). These results suggested that, upon sensing pathogen invasion, the host rapidly activated innate immune signaling networks to counteract infection whereas host energy metabolism was altered, reducing energy supply required for immune defense and thereby facilitating its own colonization and invasion.

**Figure 5:**
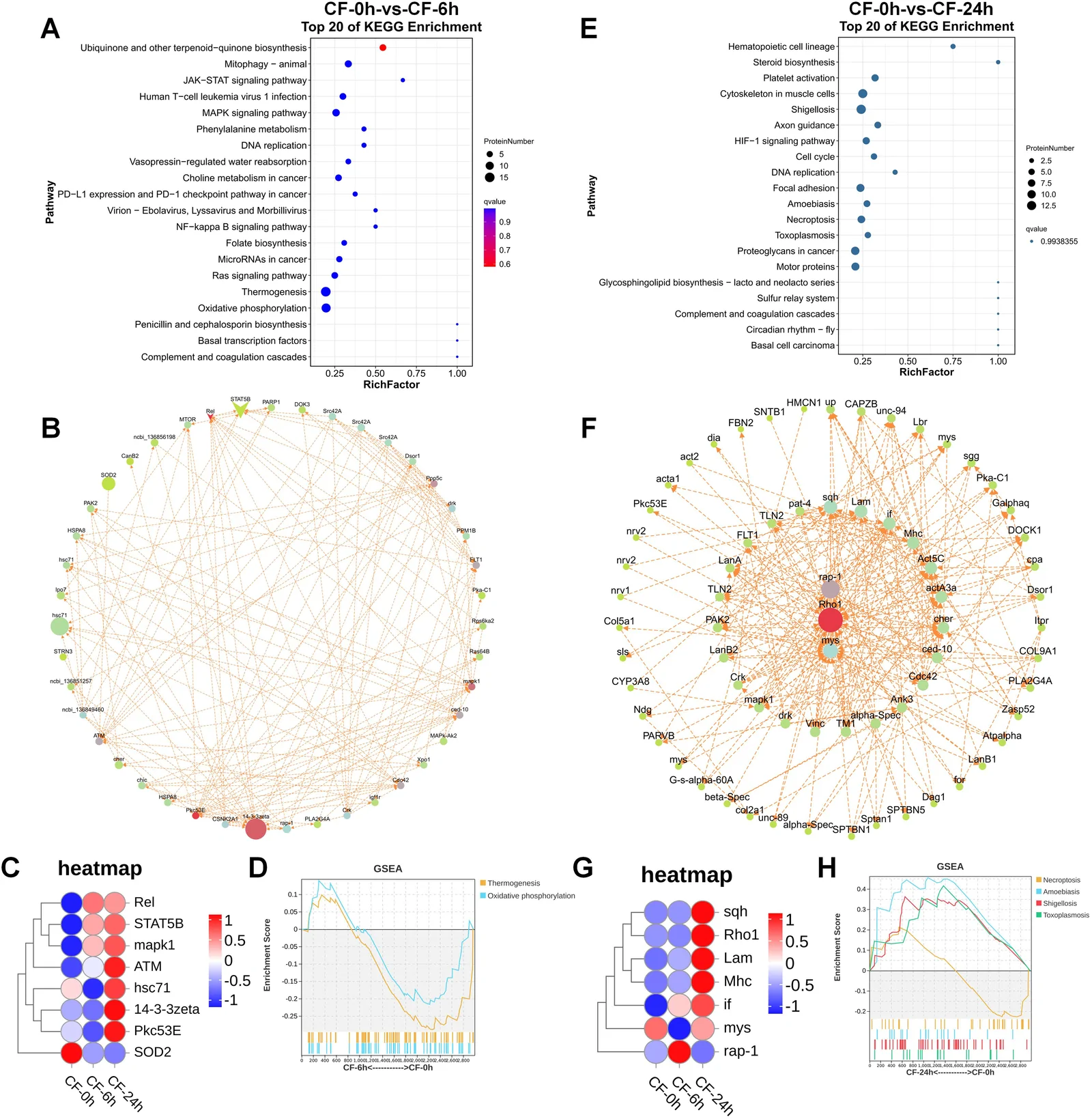
Functional analysis of DEPs in *M. rosenbergii* following *C. freundii* infection, based on KEGG enrichment analysis. **(A)** KEGG enrichment analysis of DEPs at 6 hpi with *C. freundii* infection. **(B)** PPI network analysis of DEPs involved in immune-related pathways at 6 hpi. Nodes represent proteins, with color indicating connectivity degree (nodes with deep red coloration indicate higher connectivity) and size reflecting expression level. **(C)** Heatmap visualization of core immune-related proteins. Red and blue indicate high and low relative expression, respectively. **(D)** GSEA of energy metabolism–related signaling pathways. **(E)** KEGG enrichment analysis of DEPs at 24 hpi with *C. freundii* infection. **(F)** PPI network analysis of DEPs involved in immune-related pathways at 24 hpi. Nodes represent proteins, with color indicating connectivity degree (nodes with deep red coloration indicate higher connectivity) and size reflecting expression level. **(G)** Heatmap visualization of core proteins identified in the 24 hpi network. **(H)** GSEA of tissue damage–related signaling pathways.

As the infection progressed to 24 h, KEGG pathway enrichment analysis revealed a significant shift in host response (**Figure 5E**). Multiple signaling pathways closely associated with immune cell function, such as hematopoietic cell lineage, platelet activation, and complement and coagulation cascades, were significantly enriched. Concurrently, pathways related to cellular structure and adhesion, including the cytoskeleton in muscle cells and focal adhesion, were also activated. Moreover, infection- and pathology-related pathways, such as toxoplasmosis, shigellosis, amoebiasis, and necroptosis, showed significant enrichment, indicating a transition from broad immune activation to more specialized immune regulation, structural remodeling, and pathology-associated processes. Correspondingly, the core regulatory molecules in this stage also underwent noticeable changes, with cytoskeleton- and adhesion-related proteins such as Rap1, Rho1, and mys emerging as key nodes in the network (**Figure 5F**). Expression dynamics analysis revealed that Rho1 and mys were significantly upregulated at 24 hpi whereas Rap1 was significantly upregulated at 6 h but downregulated by 24 h. Additionally, proteins, including SQH, LAM1, MHC, and IF, exhibited significant upregulation at 24 h (**Figure 5G**). Among the infection- and pathology-related pathways, most showed significant upregulation, whereas necroptosis was significantly downregulated (**Figure 5H**). Notably, in the 6 h vs 24 h comparison group, pathways involved in intracellular clearance, such as antigen processing and presentation, phagosome, autophagy, and apoptosis, were significantly downregulated whereas the actin cytoskeleton pathway was significantly upregulated (**Supplementary Figure S4**). These findings suggested that, during the later stages of infection, *C. freundii* suppressed host antigen presentation, phagocytosis, autophagy, and apoptosis while promoting cytoskeletal rearrangement, thereby facilitating immune evasion and maintaining self-survival.

These findings collectively indicate that, as the infection progressed, *C. freundii* exerted a multitargeted and time-dependent effect on the host. Early responses were dominated by activation of innate immunity, whereas later stages involved disruption of hematopoiesis, coagulation, cell adhesion, and cytoskeletal structure, coupled with systemic suppression of host intracellular clearance mechanisms. This multilayered dysregulation likely collectively contributes to immune cell dysfunction and loss of tissue integrity, providing key molecular insights into the pathogenesis of “water bubble disease” in *M. rosenbergii*.

### 3.4 Integrated analysis of transcriptomic and proteomic data

#### 3.4.1 Overview of data correlation

We integrated transcriptomic and proteomic datasets for comparative analysis to elucidate the multi-omics regulatory network underlying *C. freundii*–induced “water bubble disease” in *M. rosenbergii*. Venn diagram analysis identified 67, 106, and 66 molecules consistently differentially expressed at both transcript and protein levels in the 0 h vs 6 h, 0 h vs 24 h, and 6 h vs 24 h comparison groups, respectively (**Figures S5A–S5C**, **Supplementary Table S3**). These overlapping molecules likely represent core components of host response during infection, exhibiting coordinated regulation at transcriptional and translational levels, and may play pivotal regulatory roles in pathogen–host interaction. Nine-quadrant analysis further assessed the association between transcript and protein abundance. Pearson correlation coefficients between corresponding transcript and protein expression levels were 0.0968, 0.3058, and 0.3023 in the 0 h vs 6 h, 0 h vs 24 h, and 6 h vs 24 h comparison groups, respectively (**Figures S5D–S5F**). The progressively increasing correlation suggested that transcription and translation were largely decoupled during early infection (0 h vs 6 h) whereas coupling gradually strengthened over time, indicating an overall strengthening trend in correlation over time from decoupling toward recoupling as infection progressed.

#### 3.4.2 Host defense responses in *M. rosenbergii* induced by *C. freundii* infection

We next systematically examined the key regulatory pathways underlying the host defense response of *M. rosenbergii* to *C. freundii* infection. Nine-quadrant analysis was used to identify molecules showing consistent upregulation at both transcript and protein levels (Quadrant 3) during the early (0 h vs 6 h) and mid-to-late (0 h vs 24 h) infection stages, as well as those with unchanged transcript levels but increased protein abundance (Quadrant 6) in the 0 h vs 6 h comparison group. KEGG pathway enrichment analysis was subsequently performed on these molecular clusters (**Figures 6A–C**).

**Figure 6:**
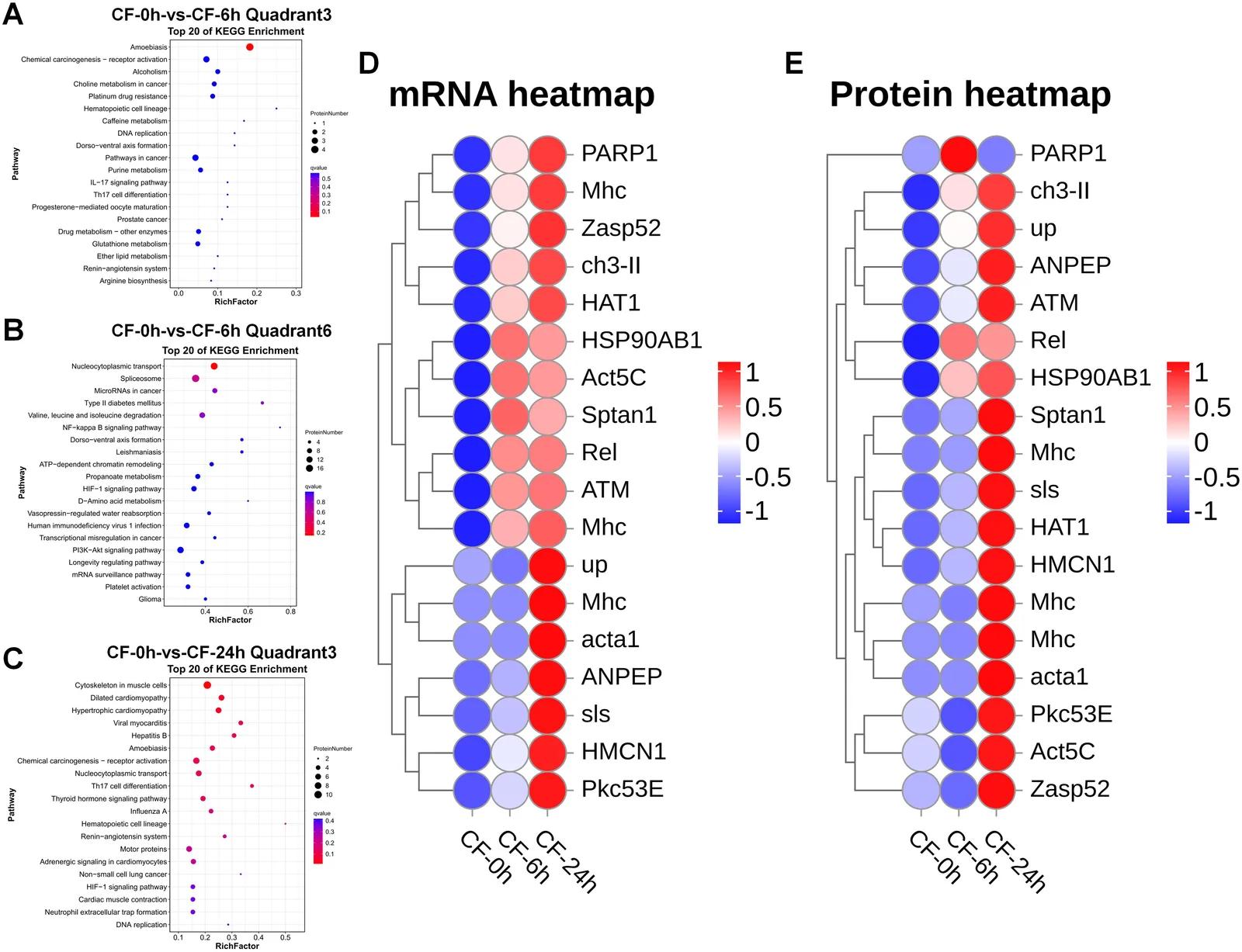
Integrated transcriptomic and proteomic analyses of host defense strategies. **(A–C)** KEGG enrichment analysis of DEGs/DEPs derived from specific quadrants of the nine-quadrant plots. Quadrant 3 (consistent upregulation at both transcript and protein levels) at 0 h vs 6 h **(A)**, Quadrant 6 (unchanged transcript but increased protein abundance) at 0 h vs 6 h **(B)**, and Quadrant 3 at 0 h vs 24 h **(C)**. **(D, E)** Heatmap analysis of mRNA **(D)** and protein **(E)** expression levels for core molecules involved in host defense across 0, 6, and 24 hpi. Red and blue indicate high and low relative expression level, respectively. Data are presented as mean ± SD (*n* = 3 biological replicates).

At 6 hpi, the Quadrant 3 cluster was significantly enriched in key inflammatory pathways, including the IL-17 signaling pathway and Th17 cell differentiation (**Figure 6A**). This indicated rapid activation of immune responses and recruitment of immune cells to establish an early inflammatory defense barrier. Simultaneously, the Quadrant 6 cluster was significantly enriched in the NF-κB signaling pathway (**Figure 6B**), a central regulator of inflammation, further corroborating that host cells actively synthesized immune effector molecules, such as cytokines, at the post-transcriptional level upon pathogen invasion.

By 24 hpi, the Quadrant 3 cluster was enriched in pathways such as neutrophil extracellular trap (NET) formation, cytoskeleton in muscle cells, dilated cardiomyopathy/hypertrophic cardiomyopathy, and cardiac muscle contraction (**Figure 6C**). These findings suggested a dual-phase host response in this stage. On the one hand, sustained immune activity was maintained through mechanisms such as extracellular trap release to capture pathogens; on the other hand, the enrichment of muscle-related structural and contractile pathways may reflect shared cytoskeletal/actin-related genes and potentially contribute to tissue damage. Collectively, these shifts implied a gradual loss of homeostatic control in the host defense system under prolonged infection.

The heatmap analysis of differentially expressed molecules within these key pathways at both transcript and protein levels (**Figures 6D, E**) revealed that most molecules were concordantly upregulated during the mid-to-late infection stages. Notably, HSP90AB1, Rel, ch3-II, and ATM exhibited sustained and significant upregulation at both molecular tiers throughout the infection timeline (**Table 2**). The qRT-PCR validation further confirmed the transcriptional upregulation of these core genes, with expression trends highly consistent with the RNA-seq data (**Supplementary Figure S6A**). These observations suggested that these molecules served as core regulators of the immune network in *M. rosenbergii*, with their persistently high expression supporting a sustained host defense response against *C. freundii*.

**Table 2:** Information on core molecules related to host defense.

Group	Quadrant	Protein/Gene ID	Proteins/Genes_Symbol	Genes_log2(fc)	Proteins_log2(fc)	Proteins/Genes_Pathway
0h vs 6h	Quadrant3	ncbi_136833196	ANPEP	1.160974677	0.735380747	Hematopoietic cell lineage
		ncbi_136831547	HSP90AB1	1.219635042	0.72297626	IL-17 signaling pathway;Th17 cell differentiation
	Quadrant6	ncbi_136825250	PARP1	0.916899103	0.496157285	NF-kappa B signaling pathway
		ncbi_136833203	Rel	0.5111364	0.675832024	NF-kappa B signaling pathway
		ncbi_136847171	ATM	0.420607507	0.456371981	NF-kappa B signaling pathway
0h vs 24h	Quadrant3	ncbi_136826282	TNNT3	1.239784271	0.504919806	Cytoskeleton in muscle cells
		ncbi_136831426	ch3-II	1.023340527	0.829195441	Neutrophil extracellular trap formation
		ncbi_136835667	SPTB	1.13358173	0.711237931	Cytoskeleton in muscle cells
		ncbi_136835968	Mhc	1.072822231	1.546774515	Cytoskeleton in muscle cells;Cardiac muscle contraction;Hypertrophic cardiomyopathy;Dilated cardiomyopathy
		ncbi_136840729	Mhc	6.144118518	1.695086876	Cytoskeleton in muscle cells;Cardiac muscle contraction;Hypertrophic cardiomyopathy;Dilated cardiomyopathy
		ncbi_136840730	Mhc	4.409129791	1.618871131	Cytoskeleton in muscle cells;Cardiac muscle contraction;Hypertrophic cardiomyopathy;Dilated cardiomyopathy
		ncbi_136836609	Titin	1.031167343	1.097900979	Cytoskeleton in muscle cells;Hypertrophic cardiomyopathy;Dilated cardiomyopathy
		ncbi_136843961	PRKCA	1.06658911	1.012888523	Neutrophil extracellular trap formation
		ncbi_136845123	LDB3	1.238540655	0.689169125	Cytoskeleton in muscle cells
		ncbi_136849270	HAT1	1.32790218	3.428262075	Neutrophil extracellular trap formation
		ncbi_136850057	ACTC1	9.232475545	10.99752694	Cytoskeleton in muscle cells;Cardiac muscle contraction;Hypertrophic cardiomyopathy;Dilated cardiomyopathy
		ncbi_136855692	ACTG1	1.312802636	0.624268438	Cytoskeleton in muscle cells;Neutrophil extracellular trap formation;Hypertrophic cardiomyopathy;Dilated cardiomyopathy
		ncbi_136856259	HMCN1	2.222392421	0.471416261	Cytoskeleton in muscle cells

#### 3.4.3 Potential immune evasion strategies of *C. freundii* during infection of *M. rosenbergii*

From the perspective of pathogen immune evasion, we further explored the molecular mechanisms underlying *C. freundii* infection in *M. rosenbergii*. Based on nine-quadrant analysis, molecules showing no transcriptional change but decreased protein abundance (Quadrant 4) in both 0 h vs 6 h and 0 h vs 24 h comparison groups, as well as those that were transcriptionally unchanged but upregulated at the protein level (Quadrant 6) in the 0 h vs 24 h comparison group, were selected for KEGG pathway enrichment analysis (**Figures 7A–C**).

**Figure 7:**
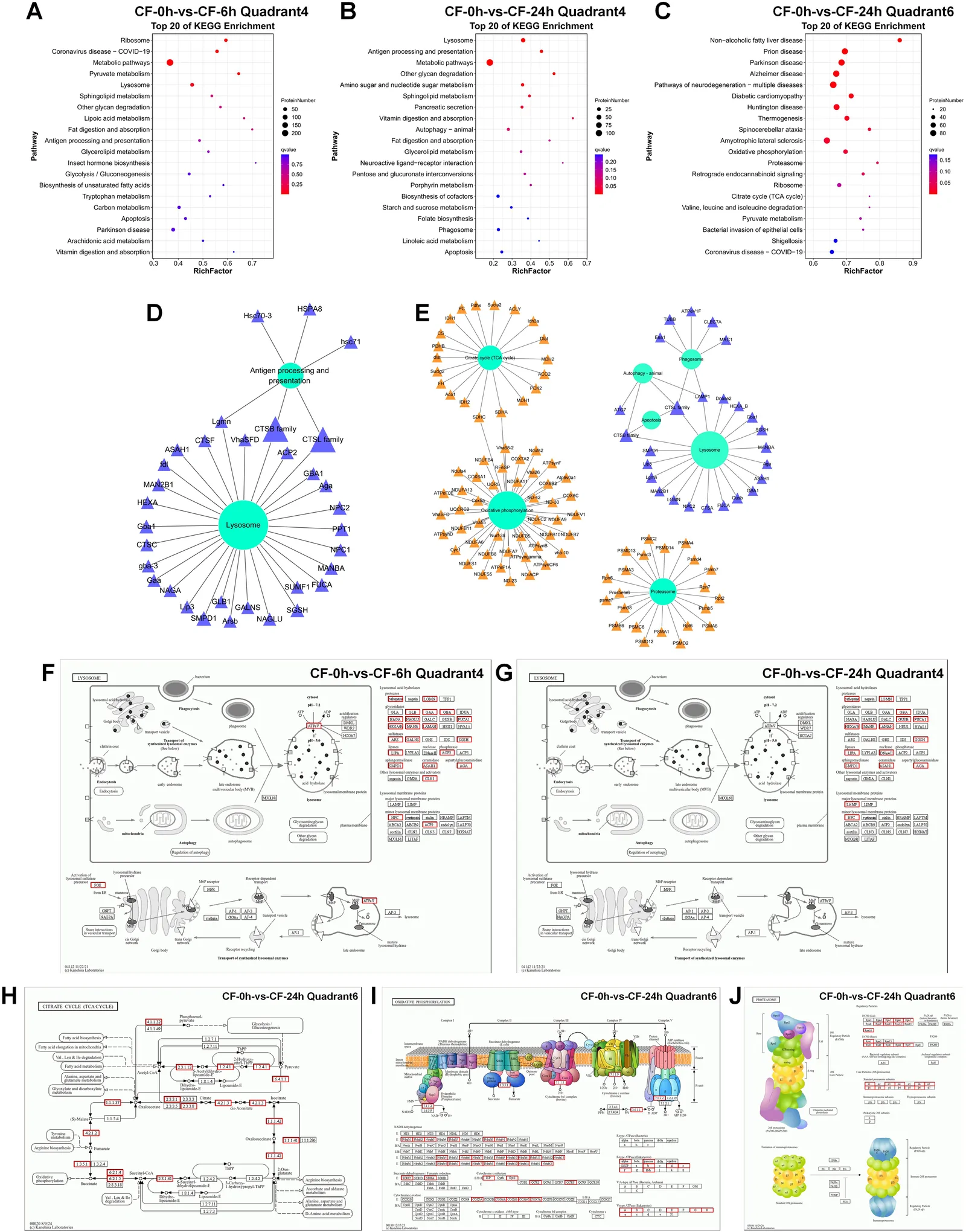
Integrated transcriptomic and proteomic analyses of pathogen immune evasion strategies. **(A–C)** KEGG enrichment analysis of DEGs/DEPs derived from specific quadrants of the nine-quadrant plots. Quadrant 4 (unchanged transcript but decreased protein abundance) at 0 h vs 6 h **(A)**, Quadrant 4 at 0 h vs 24 h **(B)**, and Quadrant 6 (unchanged transcript but increased protein abundance) at 0 h vs 24 h **(C)**. **(D, E)** PPI network analysis of core pathway molecules in the 0 h vs 6 h **(D)** and 0 h vs 24 h **(E)** comparison groups. Circles represent signaling pathways, triangles represent molecules, and larger shapes indicate higher connectivity. Purple denotes downregulation, whereas orange denotes upregulation. **(F, G)** Differentially expressed molecules involved in the lysosome signaling pathway in the 0 h vs 6 h **(F)** and 0 h vs 24 h **(G)** comparison groups. **(H–J)** Differentially expressed molecules involved in cell cycle **(H)**, oxidative phosphorylation **(I)**, and proteasome **(J)** pathways in the 0 h vs 24 h comparison group. The red frame indicates significant expression.

At 6 hpi, the Quadrant 4 cluster was significantly enriched in the antigen processing and presentation and lysosome pathways (**Figure 7A**), indicating that *C. freundii* actively interfered with host antigen presentation and lysosomal degradation functions during early infection, effectively achieving “immune invisibility” to evade recognition and clearance. Network analysis further showed that lysosome-associated proteins (e.g., CTSL family members) and heat-shock proteins within the antigen-presentation pathway occupied central regulatory positions and were significantly suppressed (**Figure 7D**).

By 24 hpi, the Quadrant 4 cluster showed significant enrichment in phagosome, autophagy (animal), and apoptosis pathways (**Figure 7B**). Core molecules, including CTSL family proteins, remained suppressed and the expression of autophagy-, apoptosis-, and phagocytosis-related molecules was broadly inhibited. qRT-PCR further verified the transcriptional inhibition of lysosome-, autophagy-, and apoptosis-related pathway genes, which was consistent with the results of transcriptomics and proteomics studies (**Supplementary Figure S6B**). This suggested that *C. freundii* reinforced suppression of host intracellular clearance mechanisms during the mid-to-late infection stages and sustained its intracellular survival by inhibiting cell-autonomous clearance mechanisms such as apoptosis. The lysosome exhibited consistent and significant suppression during infection (**Figure 7F, G**), highlighting its potential role as a key target for *C. freundii* immune evasion. In contrast, the Quadrant 6 cluster was significantly enriched in oxidative phosphorylation, citrate cycle (TCA cycle), and proteasome pathways (**Figure 7C**), with most related molecules significantly upregulated (**Figures 7E, H–J**). These findings indicated that pathogen-associated metabolic alterations and protein-degradation systems to acquire nutrients and energy, thereby gradually gaining a competitive advantage during infection and ultimately contributing to host physiological dysfunction and mortality.

## 4 Discussion

*M. rosenbergii*, a key species in global freshwater aquaculture, has been significantly constrained by various diseases, hindering its industrial development (22). A new disease known as “water bubble disease” has emerged in recent years and become a prominent bottleneck threatening the healthy and sustainable growth of the industry (3). This disease is characterized by the formation of transparent vesicles at the junction of the cephalothorax and hepatopancreas, accompanied by hepatopancreatic atrophy and necrosis, ultimately leading to reduced feeding, decreased activity, and mortality. Previous studies have identified *C. freundii* as the primary pathogen responsible for this disease (3, 4). In the present study, we confirmed *C. freundii* as the etiological agent through pathogen isolation, molecular identification, and challenge experiments, with histopathological analysis revealing progressive tissue damage including basement membrane disintegration and severe vacuolization of hepatopancreatic cells. To elucidate the molecular mechanisms underlying these pathological changes, we employed integrated transcriptomic and proteomic analyses, which revealed a sophisticated temporal interplay in which the host’s phased defense responses were systematically countered and subverted by the pathogen’s multilayered immune evasion and metabolic hijacking strategies.

The host response to *C. freundii* infection exhibited a clear temporal progression reflecting evolutionarily conserved defense strategies in crustaceans. The early phase (0–6 hpi) was characterized by a dual-pronged transcriptional program involving DNA damage repair (Fanconi anemia and nucleotide excision repair pathways) and pro-inflammatory signaling through the IL-17 and NF-κB pathways. This dual response suggests that the host rapidly recognizes *C. freundii* as a pathogen imposing both genotoxic stress and inflammatory challenge—a phenomenon increasingly recognized in host–pathogen interactions where bacterial pathogens directly damage host DNA or trigger reactive oxygen species that induce genomic instability. The IL-17 pathway, which promotes immune cell recruitment and initiates inflammatory responses, has been shown to be a key component of antibacterial immunity in both vertebrates and invertebrates (23). Notably, in crustaceans, the NF-κB pathway serves as a central regulator of innate immunity, controlling the expression of various antimicrobial peptides and cytokines upon sensing pathogen-associated molecular patterns (24, 25). The significant upregulation of Rel (an NF-κB family transcription factor) and ATM in our dataset indicates coordinated activation of immune defense and DNA damage response mechanisms, respectively. In *Drosophila*, the Relish protein, a homolog of mammalian p105/p50 and Rel, is essential for antimicrobial peptide expression in the IMD pathway (26), and ATM functions as a key kinase coordinating cellular responses to DNA double-strand breaks while also modulating immune gene expression (27). The upregulation of molecular chaperones such as HSP90AB1, which maintains protein stability during cellular stress (28, 29), and PCNA, which is indispensable for DNA replication and repair processes (30, 31), further supports the notion that the host mounts a protective response to preserve genomic and proteomic integrity. At the proteomic level, the rapid activation of JAK–STAT and MAPK signaling, alongside suppression of oxidative phosphorylation, reflects a metabolically costly reallocation of cellular resources toward immune effector production—a trade-off commonly observed in crustacean immune responses. The JAK–STAT pathway, which regulates the production of diverse effector molecules, is integral to innate immunity in insects and crustaceans, and the upregulation of STAT5B underscores its conserved role in transmitting cytokine signals and regulating immune gene transcription (32–34).

The aberrant activation of pathways related to muscle structure and function pathways—including dilated cardiomyopathy, cardiac muscle contraction, and actin cytoskeleton regulation, at 24 hpi warrants particular attention. In crustaceans, the actin cytoskeleton serves as a primary force-generating machinery that regulates cellular morphology, adhesion, and barrier integrity (35, 36). Pathogens frequently exploit host cytoskeletal components to facilitate infection: for example, WSSV utilizes the actin cytoskeleton for intracellular transport of viral nucleocapsids, while bacterial pathogens can manipulate cytoskeletal proteins to promote host-cell invasion (37). The Rho family of small GTPases, including RhoA, are pivotal regulators of cytoskeletal dynamics and play essential roles in crustacean antibacterial immunity (38, 39). The significant upregulation of cytoskeletal regulators such as Rho1 and mys observed in our study therefore suggests active and potentially dysregulated cytoskeletal remodeling during infection, which may compromise the structural integrity of the hepatopancreas and surrounding tissues. This interpretation is consistent with previous histopathological observationso of crustacean infections. In *P. vannamei* infected with *V. parahaemolyticus*, disrupted hepatopancreatic tubules accompanied by diffuse interstitial edema have been reported (40). Similarly, recent surveillance studies in penaeid shrimp have consistently associated bacterial infections with muscle necrosis and hemocyte infiltration (41); while muscular changes, including myofibrillar disorganization and necrotic lesions, have been documented in *M. rosenbergii* infected with *P. aeruginosa* (42). The significant enrichment of pathways related to dilated cardiomyopathy and cardiac muscle contraction further suggests impaired myocardial contractility. In the open circulatory system of crustaceans, cardiac activity is regulated by complex neurogenic and neurohormonal mechanisms, and compromised cardiac performance could impair hemolymph circulation and vascular integrity, leading to increased permeability and fluid extravasation (43). Collectively, these results suggest that the “water bubble” phenotype may be associated with pathogen-induced cytoskeletal dysregulation, muscle tissue damage, and impaired cardiac contractility, potentially contributing to interstitial fluid accumulation at the cephalothorax–hepatopancreas junction. The enrichment of cardiac muscle contraction and cardiomyopathy pathways in the hepatopancreas may partly reflect shared cytoskeletal/actin-related genes. This transition from immune-focused defense to structural–pathological remodeling may represent a key tipping point in disease progression.

Our integrated analysis further reveals that *C. freundii* employed a time-dependent, multitarget strategy to subvert host defenses. During early infection, the pathogen achieves immune evasion by specifically downregulating the host’s antigen processing and presentation machinery and lysosomal function, particularly through suppression of CTSL family proteases. Lysosomal proteases, especially cathepsins, are crucial for antigen processing, and their inhibition directly impairs the initiation of immune responses (44, 45). By 24 hpi, this strategy expands into a broad-spectrum blockade of intracellular clearance mechanisms, including phagosome maturation, autophagy, and apoptosis—all of which are essential for eliminating intracellular pathogens and restricting their spread (46). The coordinated suppression of these pathways effectively disables the host’s cell-autonomous defense systems, allowing sustained intracellular survival of the pathogen. Concurrently, while shutting down host defenses, *C. freundii* appears to co-opt host resources through upregulation of the proteasome and central energy metabolism pathways (oxidative phosphorylation, TCA cycle), suggesting a pathogen-associated metabolic alterations that diverts host ATP and nutrients to fuel its own replication. This dual tactic of defense suppression and resource piracy underscores a highly adapted parasitic strategy that enables the pathogen to gain a competitive advantage during infection.

In summary, our integrated multi-omics analysis revealed a bidirectional and dynamic interplay underlying *C. freundii*–induced “water bubble disease” in *M. rosenbergii*. During early infection, the host launched an immune defense centered on IL-17 and NF-κB signaling, accompanied by activation of DNA repair pathways. Concurrently, *C. freundii* employed a stealth strategy, suppressing antigen presentation and hijacking host energy metabolism, to evade detection and establish infection. As the infection progressed to the late stage, the host response shifted from protective immunity toward pathological damage. This phase was marked by persistent DNA repair dysfunction, NET formation, and cytoskeletal disruption. In parallel, the pathogen intensified its offensive strategy by broadly inhibiting phagocytosis and apoptosis, compromising cellular integrity and promoting host metabolic resources. Moreover, the transcriptomic–proteomic correlation analysis further indicated a temporal shift from post-transcriptional decoupling to recoupling, reflecting stage-specific regulatory adaptations within the host (**Figure 8**). Our findings provide a systematic framework for understanding the pathogenesis of “water bubble disease” in *M. rosenbergii* and identify potential targets, including lysosomal function, cytoskeletal stability, and metabolic pathways, for developing targeted interventions to mitigate disease onset and progression. However, this study is based on transcriptomic and proteomic correlation analysis and cannot fully establish causality; therefore, further functional experimental validation from both pathogen and host perspectives is needed in future studies.

**Figure 8:**
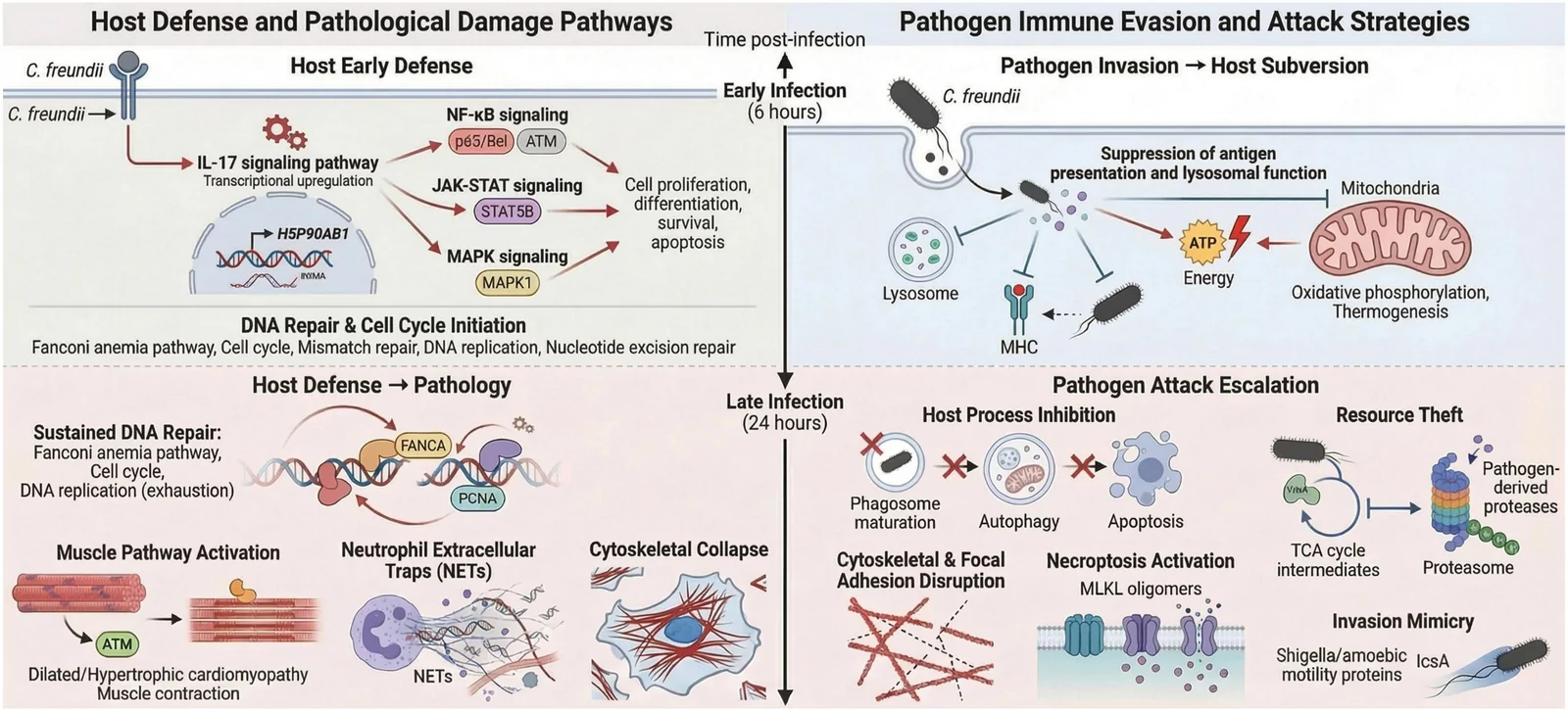
A bidirectional dynamic mechanism model of *C. freundii–*induced “water bubble disease” in *M. rosenbergii*. During early infection (6 h), the host initiates immune defense through signaling pathways such as IL-17 and NF-κB and activates DNA repair systems. Concurrently, the pathogen achieves immune evasion by suppressing antigen presentation and hijacking energy metabolism. As infection progresses to the late stage (24 h), the host response shifts toward pathological damage, characterized by persistent DNA repair abnormalities, NET formation, and cytoskeletal disruption. Simultaneously, the pathogen launches a comprehensive attack through multiple strategies, including the suppression of phagocytosis and apoptosis, disruption of cellular structures, and exploitation of metabolic resources. Transcriptomic–proteomic correlation analysis further revealed a temporal transition from “decoupling” to “recoupling” during infection, indicating that the host adopts distinct, stage-specific molecular response strategies during the course of infection. Red arrows indicate activation/upregulation; blue arrows indicate inhibition/suppression. This model was generated using Citexs (https://www.citexs.com/).

## Data Availability

The datasets presented in this study can be found in online repositories. The names of the repository/repositories and accession number(s) can be found below: https://www.ncbi.nlm.nih.gov/, PRJNA1471540.

## Supplementary material

Supplementary Table S1 Primers used in this study.

Supplementary Table S2 Basic information on proteomic sequencing.

Supplementary Table S3 Molecular quantity information in the nine quadrants.
