# Supplementary material for: Integrated transcriptomic and proteomic analyses of the mechanisms of Citrobacter freundii–induced ‘‘water bubble disease’’ in Macrobrachium rosenbergii
Source: Front Immunol. 2026 Oct 22;17:1894276. doi: 10.3389/fimmu.2026.1894276 (PMC13735598; doi:10.3389/fimmu.2026.1894276)
Supplement: Supplementary file 1 [file Table1.docx]

| Sample | RawDatas | Clean_Reads(%) | Unmapped_Reads(%) | Total_Mapped(%) | exon | Refer_Genes | Novel_Genes | Total_Genes | sequenced_Total_Genes(%) |
| --- | --- | --- | --- | --- | --- | --- | --- | --- | --- |
| CF-0h-1 | 42215130 | 42055520 (99.62%) | 39256176 ( 93.34% ) | 36764699 (93.65%) | 30919239 (84.10%) | 23057 | 1778 | 24835 | 13328 (53.67%) |
| CF-0h-2 | 42092010 | 41948824 (99.66%) | 39139388 ( 93.30% ) | 36661951 (93.67%) | 30813070 (84.05%) | 23057 | 1778 | 24835 | 13123 (52.84%) |
| CF-0h-3 | 41228690 | 41082076 (99.64%) | 38289008 ( 93.20% ) | 35760250 (93.40%) | 29973862 (83.82%) | 23057 | 1778 | 24835 | 13080 (52.67%) |
| CF-6h-1 | 42256746 | 42090498 (99.61%) | 38263730 ( 90.91% ) | 35626615 (93.11%) | 29371539 (82.44%) | 23057 | 1778 | 24835 | 14518 (58.46%) |
| CF-6h-2 | 44604162 | 44443644 (99.64%) | 41590700 ( 93.58% ) | 38937298 (93.62%) | 32330555 (83.03%) | 23057 | 1778 | 24835 | 14971 (60.28%) |
| CF-6h-3 | 44576750 | 44409134 (99.62%) | 39565280 ( 89.09% ) | 36871202 (93.19%) | 30299655 (82.18%) | 23057 | 1778 | 24835 | 14357 (57.81%) |
| CF-24h-1 | 44790784 | 44603468 (99.58%) | 39779098 ( 89.18% ) | 36947997 (92.88%) | 30581642 (82.77%) | 23057 | 1778 | 24835 | 14901 (60.00%) |
| CF-24h-2 | 43250352 | 43083028 (99.61%) | 39224964 ( 91.05% ) | 36628109 (93.38%) | 30462231 (83.17%) | 23057 | 1778 | 24835 | 15149 (61.00%) |
| CF-24h-3 | 45609474 | 45485020 (99.73%) | 40670628 ( 89.42% ) | 37698008 (92.69%) | 31180766 (82.71%) | 23057 | 1778 | 24835 | 14885 (59.94%) |
