# Supplementary material for: Integrated transcriptomic and proteomic analyses of the mechanisms of Citrobacter freundii–induced ‘‘water bubble disease’’ in Macrobrachium rosenbergii
Source: Front Immunol. 2026 Oct 22;17:1894276. doi: 10.3389/fimmu.2026.1894276 (PMC13735598; doi:10.3389/fimmu.2026.1894276)
Supplement: Supplementary file 2 [file Table2.docx]

| Group | Quadrant | Protein/Gene ID | Proteins/Genes_Symbol | Genes_log2(fc) | Proteins_log2(fc) | Proteins/Genes_Pathway |
| --- | --- | --- | --- | --- | --- | --- |
| 0h vs 6h | Quadrant3 | ncbi_136833196 | ANPEP | 1.160974677 | 0.735380747 | Hematopoietic cell lineage |
|  |  | ncbi_136831547 | HSP90AB1 | 1.219635042 | 0.72297626 | IL-17 signaling pathway;Th17 cell differentiation |
|  | Quadrant6 | ncbi_136825250 | PARP1 | 0.916899103 | 0.496157285 | NF-kappa B signaling pathway |
|  |  | ncbi_136833203 | Rel | 0.5111364 | 0.675832024 | NF-kappa B signaling pathway |
|  |  | ncbi_136847171 | ATM | 0.420607507 | 0.456371981 | NF-kappa B signaling pathway |
| 0h vs 24h | Quadrant3 | ncbi_136826282 | TNNT3 | 1.239784271 | 0.504919806 | Cytoskeleton in muscle cells |
|  |  | ncbi_136831426 | ch3-II | 1.023340527 | 0.829195441 | Neutrophil extracellular trap formation |
|  |  | ncbi_136835667 | SPTB | 1.13358173 | 0.711237931 | Cytoskeleton in muscle cells |
|  |  | ncbi_136835968 | Mhc | 1.072822231 | 1.546774515 | Cytoskeleton in muscle cells;Cardiac muscle contraction;Hypertrophic cardiomyopathy;Dilated cardiomyopathy |
|  |  | ncbi_136840729 | Mhc | 6.144118518 | 1.695086876 | Cytoskeleton in muscle cells;Cardiac muscle contraction;Hypertrophic cardiomyopathy;Dilated cardiomyopathy |
|  |  | ncbi_136840730 | Mhc | 4.409129791 | 1.618871131 | Cytoskeleton in muscle cells;Cardiac muscle contraction;Hypertrophic cardiomyopathy;Dilated cardiomyopathy |
|  |  | ncbi_136836609 | Titin | 1.031167343 | 1.097900979 | Cytoskeleton in muscle cells;Hypertrophic cardiomyopathy;Dilated cardiomyopathy |
|  |  | ncbi_136843961 | PRKCA | 1.06658911 | 1.012888523 | Neutrophil extracellular trap formation |
|  |  | ncbi_136845123 | LDB3 | 1.238540655 | 0.689169125 | Cytoskeleton in muscle cells |
|  |  | ncbi_136849270 | HAT1 | 1.32790218 | 3.428262075 | Neutrophil extracellular trap formation |
|  |  | ncbi_136850057 | ACTC1 | 9.232475545 | 10.99752694 | Cytoskeleton in muscle cells;Cardiac muscle contraction;Hypertrophic cardiomyopathy;Dilated cardiomyopathy |
|  |  | ncbi_136855692 | ACTG1 | 1.312802636 | 0.624268438 | Cytoskeleton in muscle cells;Neutrophil extracellular trap formation;Hypertrophic cardiomyopathy;Dilated cardiomyopathy |
|  |  | ncbi_136856259 | HMCN1 | 2.222392421 | 0.471416261 | Cytoskeleton in muscle cells |
