# Supplementary material for: Integrated transcriptomic and proteomic analyses of the mechanisms of Citrobacter freundii–induced ‘‘water bubble disease’’ in Macrobrachium rosenbergii
Source: Front Immunol. 2026 Oct 22;17:1894276. doi: 10.3389/fimmu.2026.1894276 (PMC13735598; doi:10.3389/fimmu.2026.1894276)
Supplement: Supplementary file 3 [file Table3.docx]

| **Primer name** | **Sequence (5’-3’)** |
| --- | --- |
| LDB3-F-qRT | AGATCGGCGGGAAGGACG |
| LDB3-R-qRT | TCGCAGCAAGTGAGGTGG |
| HSP90-F-qRT | TTACTCCGCTACCACACC |
| HSP90-R-qRT | GACAACCTCAAATCCACG |
| HMCN1-F-qRT | TAGCCCCTCCGATTGTTA |
| HMCN1-R-qRT | GCAGGACGGTCATGTACC |
| HAT1-F-qRT | TCAACGGAAACCCTCAGT |
| HAT1-R-qRT | AGTCTCGTACCCGCACAA |
| ch3-II-F-qRT | AGATCGCCCTGTCCATCC |
| ch3-II-R-qRT | TGTTTCTGCCTTTCCCTA |
| ATM-F-qRT | GTCATCGCTTCCTCCCATC |
| ATM-R-qRT | CGCAACATTTTCCCACATT |
| ANPEP-F-qRT | TAGAGGACCAGGAGGGTT |
| ANPEP-R-qRT | TTTTGGCAGAGGATAAGG |
| ACTG1-F-qRT | GATGGCGTCTCCCACACT |
| ACTG1-R-qRT | TGAACCTCTCGTTACCGA |
| ACTC1-F-qRT | TGGGACGACATGGAAAAG |
| ACTC1-R-qRT | GCCAGAGGCATAGAGGGA |
| TNNT3-F-qRT | GGCAAACACCCACCCAAA |
| TNNT3-R-qRT | GTCGCTCGCCGAACCACT |
| Titin-F-qRT | CGCAATGATAAACCACTT |
| Titin-R-qRT | TTCGGTAACCATAAGAGG |
| SPTB-F-qRT | ACCTGAAGGAGCGAGTGT |
| SPTB-R-qRT | CGGCGGAATAGATGTAAC |
| Rel-F-qRT | TATGGAATGATGTCCCCTG |
| Rel-R-qRT | TGTTGTTGTTGATGCTGCT |
| PRKCA-F-qRT | ATTGGTGGGCTTACGGTG |
| PRKCA-R-qRT | TCGCTTCTGAGGCTGCTT |
| PARP1-F-qRT | ATGAAGAAGACAAGAGTGC |
| PARP1-R-qRT | CAAGTTTATAATGGGAATG |
| Mhc-40730-F-qRT | GAAGCCAACCGAACACTC |
| Mhc-40730-R-qRT | TGCACTCCTCGTCAGCAA |
| Mhc-40729-F-qRT | TGCACATGGGTGAAATGA |
| Mhc-40729-R-qRT | ACCAAGAACCTGAAGACG |
| Mhc-35968-F-qRT | ACTTCAGGTCCGGTTAGA |
| Mhc-35968-R-qRT | CTCCTTGATGCGTCTCTC |
| LCP2-31388-F-qRT | CTTTCCTGCTATCTCGCT |
| LCP2-31388-R-qRT | TTTGCTGCCTTGCTTACT |
| LCP2-31396-F-qRT | GAGGGAATGGAATTCGTT |
| LCP2-31396-R-qRT | CGCTGTTTTCTGTTGAGC |
| LCP3-31383-F-qRT | GAAGCCAACTACCCCTAC |
| LCP3-31383-R-qRT | AACCGACTGCCAACACAG |
| LCP2-31719-F-qRT | AGTGTGTGCGTCGATGCT |
| LCP2-31719-R-qRT | TTGTTCCTGGTGGGCTTC |
| Cys-49834-F-qRT | TGGGGTGAGGCTGGATAT |
| Cys-49834-R-qRT | CAAAGGGTTGGCATAGGA |
| LCP1-33362-F-qRT | ACCCGCCAAAGGAAATAA |
| LCP1-33362-R-qRT | CCAGCGTTGCCCCAGTCT |
| LCP2-31393-F-qRT | TAATGAACGCTTCCAAAA |
| LCP2-31393-R-qRT | GACCAGCAAGATCCACAC |
| CTSB-45853-F-qRT | AAAATCCCAACACCCCCA |
| CTSB-45853-R-qRT | CCATAGCACCCGAGACCA |
| CTSB-50829-F-qRT | TAAAGGGACTCTTGGGTG |
| CTSB-50829-R-qRT | AATCGGTGTCGTTGTTGT |
| CTSB-45840-F-qRT | TCAGGCGACTGTGGGTTT |
| CTSB-45840-R-qRT | CCTTCAATATGGTGGGGG |
| CTSB-50810-F-qRT | TCAACAAGAATCTCCCGA |
| CTSB-50810-R-qRT | TTACCTCCACTGCTCCAA |
| CTSB-50826-F-qRT | GTTGGAGCAGTCAGTGTC |
| CTSB-50826-R-qRT | TTCTTTGGAGTTGTAGCG |
| LAMP1-F-qRT | CACTACCCTACCCCCAGA |
| LAMP1-R-qRT | GTGACAGCCCCTTCAATC |
| SMPD1-F-qRT | CGGCTTGATGTTAGGGGG |
| SMPD1-R-qRT | CCAGGGTACGGAGAGGGA |
| SGSH-F-qRT | GCCTCTCTTGGTGTCCTC |
| SGSH-R-qRT | GCCCGTTCTAACTCCTGC |
| ATG7-F-qRT | GATGTCTGCTTTTAGGGG |
| ATG7-R-qRT | TCAATGAGAGATTCGCCT |
| EF-1α-F-qRT | ATGTCATGGTGGAAGAAGAG |
| EF-1α-R-qRT | AAAGTTGACCACCATACCAG |
