# Supplementary material for: Integrated transcriptomic and proteomic analyses of the mechanisms of Citrobacter freundii–induced ‘‘water bubble disease’’ in Macrobrachium rosenbergii
Source: Front Immunol. 2026 Oct 22;17:1894276. doi: 10.3389/fimmu.2026.1894276 (PMC13735598; doi:10.3389/fimmu.2026.1894276)
Supplement: Supplementary file 4 [file Table4.docx]

| Type | Precursors | Peptides | Protein_Groups | Proteins | GO | KEGG | KOG | Annotation proteins | Without annotation proteins |
| --- | --- | --- | --- | --- | --- | --- | --- | --- | --- |
| Numbers | 17592 | 15252 | 2910 | 2975 | 2677 | 1454 | 2269 | 2732 | 243 |
