# Supplementary material for: Integrated transcriptomic and proteomic analyses of the mechanisms of Citrobacter freundii–induced ‘‘water bubble disease’’ in Macrobrachium rosenbergii
Source: Front Immunol. 2026 Oct 22;17:1894276. doi: 10.3389/fimmu.2026.1894276 (PMC13735598; doi:10.3389/fimmu.2026.1894276)
Supplement: Supplementary file 5 [file Table5.docx]

| Type | CF-0h-vs-CF-6h | | |  | CF-0h-vs-CF-24h | | |  | CF-6h-vs-CF-24h | | |
| --- | --- | --- | --- | --- | --- | --- | --- | --- | --- | --- | --- |
|  | DEP_Down | NDEP | DEP_Up |  | DEP_Down | NDEP | DEP_Up |  | DEP_Down | NDEP | DEP_Up |
| DEG_Up | 70 | 96 | 97 |  | 34 | 65 | 252 |  | 11 | 28 | 136 |
| NDEG | 806 | 1081 | 588 |  | 362 | 702 | 1323 |  | 346 | 828 | 1392 |
| DEG_Down | 61 | 39 | 17 |  | 66 | 19 | 57 |  | 44 | 45 | 51 |
